# Sub-Antarctic subtidal and intertidal macroalgae in rocky ecosystems of Inútil Bay, Tierra del Fuego in Southern Patagonia

**DOI:** 10.3897/BDJ.14.e183377

**Published:** 2026-04-20

**Authors:** Julieta Kaminsky, Mauricio Palacios, Mariano Rodríguez, Mathias Hüne

**Affiliations:** 1 CADIC-CONICET, Ushuaia, Argentina CADIC-CONICET Ushuaia Argentina; 2 WHOI, Woods Hole, United States of America WHOI Woods Hole United States of America; 3 Fundación Rewilding Chile, Puerto Varas, Chile Fundación Rewilding Chile Puerto Varas Chile; 4 Centro de Investigación de Ecosistemas de la Patagonia (CIEP), Coyhaique, Chile Centro de Investigación de Ecosistemas de la Patagonia (CIEP) Coyhaique Chile; 5 Instituto de Ciencias Polares, Ambiente y Recursos Naturales, Universidad Nacional de Tierra del Fuego, Ushuaia, Argentina Instituto de Ciencias Polares, Ambiente y Recursos Naturales, Universidad Nacional de Tierra del Fuego Ushuaia Argentina

**Keywords:** benthic inventory, Magellan Strait, occurrence dataset, photo-quadrats, scientific diving

## Abstract

**Background:**

The sub-Antarctic region is notable for its high macroalgal diversity. Although a number of studies have documented the species richness of macroalgae along the Chilean sub-Antarctic coast, many regions remain underexplored, with little to no available species inventories or ecological assessments.

**New information:**

This study aims to expand current knowledge of macroalgal species richness in the intertidal and subtidal rocky environments in Inútil Bay, a sub-Antarctic area of Tierra del Fuego (Chile). A total of 72 macroalgal taxa were identified: 32 in intertidal and 58 in subtidal habitats. A comprehensive species list is provided, accompanied by photographs of representative macroalgal species.

## Introduction

Benthic marine macroalgae are distributed in a varied range of rocky coastal ecosystems around the world, both in intertidal and subtidal zones. Environmental factors, such as resource availability (including rocky substrate, light and nutrients), tidal amplitude, wave exposure, air and water temperature, play a crucial role in regulating macroalgae distribution and abundances (e.g. Wallenstein et al. (2007), Deregibus et al. (2016)). In these coastal ecosystems, macroalgae can play a key ecological role. They can modify the structure and physical conditions of habitats, acting as ecosystem engineers, enhancing structurally complex habitats that support high biodiversity (e.g. Teagle et al. (2017), Gutiérrez et al. (2019), Gutiérrez et al. (2023)). Their primary production supports rich food webs and they have the potential to contribute significantly to blue carbon sequestration through the transport and burial of algae biomass in the deep ocean (Queirós et al. 2019, Filbee-Dexter et al. 2024). Furthermore, macroalgal communities can serve as indicators of environmental health: changes in species composition, coverage or biomass can be used as a signal of pollution or other anthropogenic disturbances (Kaminsky et al. 2024). Alternative stable states may emerge within these assemblages; thus, improving the taxonomic resolution of macroalgal surveys is critical for advancing ecosystem monitoring. To achieve this, it is essential to broaden our understanding of macroalgal species richness in understudied regions such as sub-Antarctic coastal ecosystems and to develop comprehensive collections of specimens – supplemented by photographs and in situ records – to better capture species' morphological variability across life stages and environmental conditions. A more complete taxonomic approach can improve comparisons across ecosystems, provide information for conservation priorities and help detect invasive species.

The sub-Antarctic region is notable for its high macroalgal diversity (Ramírez 2010, Liuzzi et al. 2011). A number of studies have documented the species richness of macroalgae along the Chilean sub-Antarctic coast, including foundational works by Skottsberg (1941), Papenfuss (1964), Santelices and Ojeda (1984), Ramírez (2010), Mansilla et al. (2013), Cárdenas and Montiel (2015), Mystikou et al. (2016), Palacios (2018), Ojeda et al. (2019), Mansilla et al. (2020), Marambio et al. (2020), Jofre et al. (2021), , and Rodríguez et al. (2024). Although intertidal environments can be more frequently studied due to easier access, many sub-Antarctic regions remain underexplored, with little to no available species inventories or ecological assessments. Some studies have characterised species richness, zonation patterns and spatial-temporal variation in biomass and richness in intertidal environments (e.g. Ojeda et al. (2019) in Bahía Róbalo, Beagle Channel). Regarding subtidal macroalgae, historical limitations in sampling – relying on bycatch or drift algae – meant that early descriptions of subtidal flora were often incomplete. In recent decades, the use of Self-Contained Underwater Breathing Apparatus (SCUBA) diving has enabled more accurate surveys in these environments (e.g. Cárdenas and Montiel (2015), Friedlander et al. (2020), Kaminsky et al. (2024)). This method allows for careful specimen collection and the integration of photographic records that are essential for both taxonomic validation and environmental characterisation. This is particularly important since herbarium specimens often lose natural colouration and shape, making field identification more difficult. Amongst scientific diving techniques, the use of transects with census in situ and photo-quadrats has further enhanced data quality and spatial resolution. While there have been increasing efforts to document benthic biodiversity, most studies have focused on invertebrates and fish, with macroalgal communities remaining under-described. Recently, methods like the Roving Diver Technique have been applied to photograph and collect macroalgae in situ for identification and open-access data sharing on citizen-science platforms — offering a valuable resource for future monitoring (Bravo et al. 2023). This has proven useful in remote locations such as Inútil Bay in the sub-Antarctic Magellan Region, which, despite its ecological importance and extensive kelp forests and intertidal habitats, remains largely understudied due to logistical difficulties.

**Figure 1.**
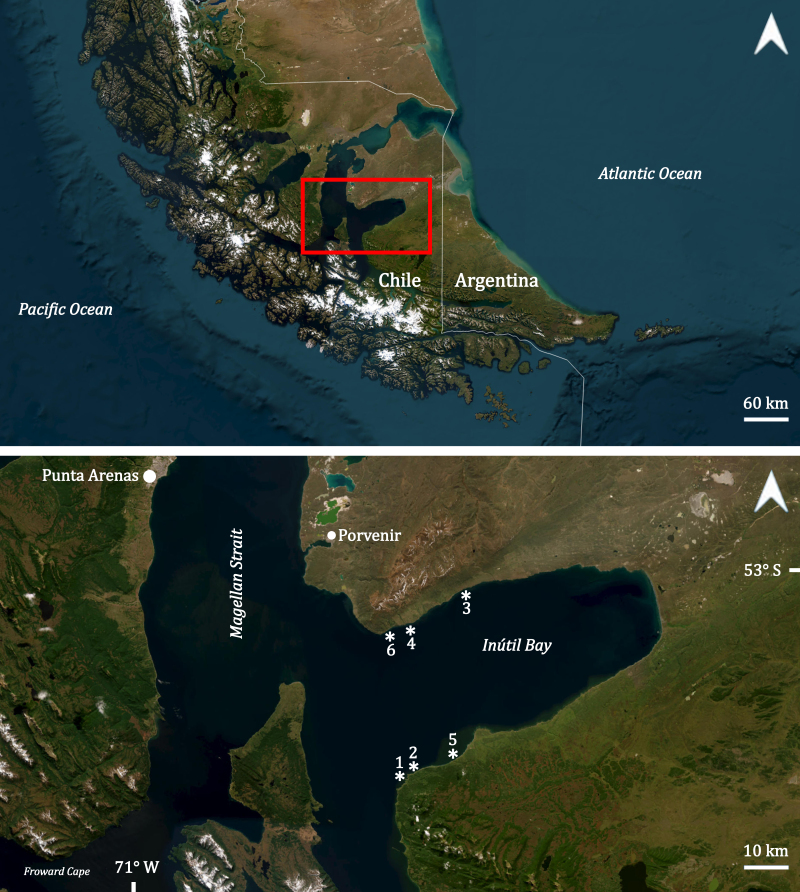
Location of Inútil Bay, with sampling sites indicated in the northern and southern regions of the Bay. Satellite images were obtained from ESRI Satellite in QGIS (version 3.40.3).

This study aims to expand current knowledge of macroalgal species richness in the intertidal and subtidal rocky environments in an area of Tierra del Fuego (Chile), which has been identified as an Area of High Marine Conservation Value in Southern Patagonia (Vila et al. 2010), such as Inútil Bay, where intertidal and subtidal ecosystems of benthic macroalgae had never been explored (Fig. 1). As part of the Fundación Rewilding Chile’s Bahía Inútil Marine Protected Area Project, field surveys were conducted along both the northern and southern coasts of the Bay in March 2025. These efforts seek to document the biodiversity of these ecosystems and generate a baseline for future monitoring that includes macroalgal diversity (Pérez et al., *in prep*). This study includes a checklist of species observed in both intertidal and subtidal zones, with particular attention given to understorey algae within kelp forests. By integrating findings across habitats, this work contributes to filling biogeographic knowledge gaps, supporting conservation planning and improving ecological understanding of sub-Antarctic coastal ecosystems.

## Project description

### Title

Fundación Rewilding Chile: Bahía Inútil expedition.

### Personnel

Julieta Kaminsky, Mauricio Palacios, Mariano Rodríguez, Ernesto Davis, Daniel Pérez, Jonathan Poblete, Mathias Hüne

### Study area description

Inútil Bay is located in the Magallanes Region, at the southern tip of the Tierra del Fuego archipelago in South America (Fig. 1). In this sub-Antarctic region, there is a connection between the Pacific and Atlantic Oceans through the transport of low-salinity waters via the Strait of Magellan (Brun et al. 2020). Inútil Bay presents a mixed semi-diurnal tidal regime with an average amplitude of 4 m (Garces et al., *in prep*), which plays an important role in structuring intertidal and subtidal habitats. This subpolar area is characterised by highly seasonal environmental conditions, especially regarding temperature and light availability for photosynthesis in near-surface waters (Iriarte et al. 2014). Inside the Bay, prevailing westerly winds dominate and can reach sustained intensities of 25–30 kt on average annually, accompanied by average annual temperatures of approximately 6.3°C (INIA 2025). During this expedition, conducted at the end of the austral summer (March 2025), the average seawater temperature was 10.08°C (± 0.69) and salinity was 28.45 (± 0.61) psu. Seawater temperature and salinity were measured with a HANNA HI 98194 multiparametric sensor.

### Design description

**Figure 2.**
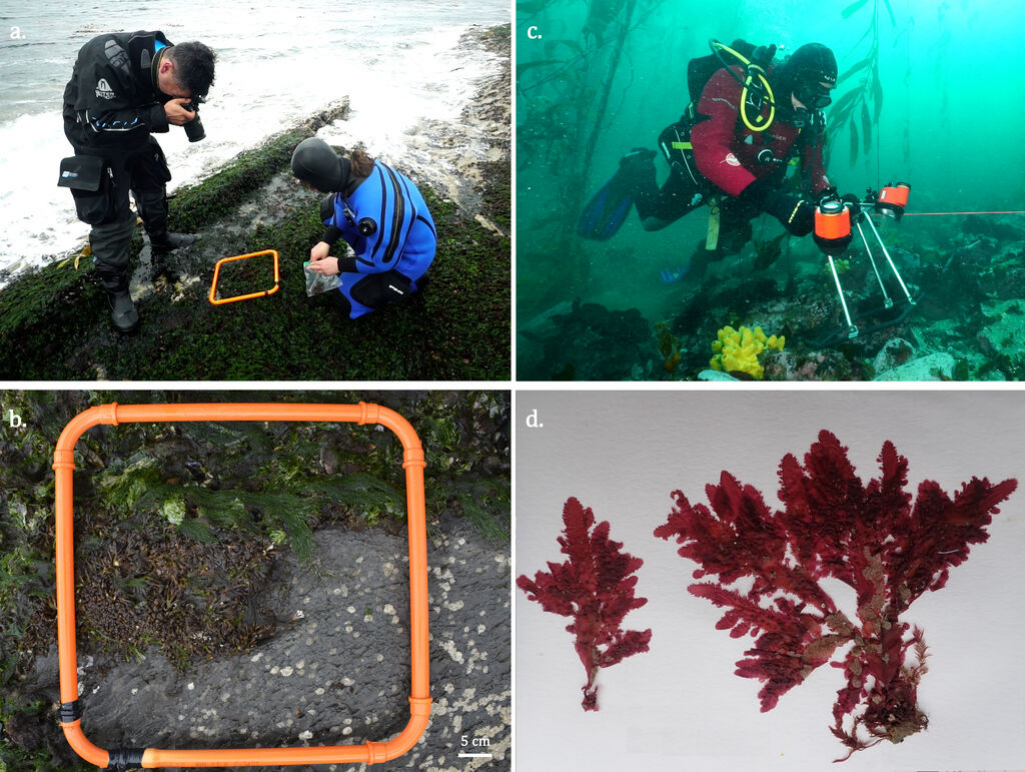
Sampling methods in Inútil Bay. Photo-quadrats in the intertidal (**a**, **b**) and subtidal environments (**c**); example of a herbarium specimen of *Cladodonta lyallii* (**d**). Photographs: Mariano Rodriguez (a and c), Mauricio Palacios (b), Julieta Kaminsky (d).

To describe benthic biodiversity in Inútil Bay, six sampling sites were selected: three along the northern coast and three along the southern coast of the Bay (Fig. 1, Table 1). To assess intertidal species richness, 25-metre transects were established parallel to the coastline at each site across three tidal levels — high, mid- and low. Along each transect, ten 25 cm × 25 cm photo-quadrats were taken at each intertidal level (totalling 50 m² per transect and 30 quadrats per site; Fig. 2a and b). To study subtidal species assemblages, two 25-metre SCUBA transects were conducted at each site within kelp forests parallel to the coastline and extending 1 m on either side. Twenty 25 cm × 25 cm photo-quadrats were recorded along each transect (50 m² area per transect; 40 quadrats per site; Fig. 2c). Additionally, macroalgal specimens were collected to support species identification from the photo-quadrats. Specimen collection was only possible at sites 2 through 5 due to meteorological constraints (especially strong winds) that made fieldwork very complex. These samples were identified, based on thallus anatomy and morphology using regional taxonomic references. Taxonomic identification was performed, based on external morphological characteristics, with specimens classified to the lowest possible taxonomic level. Specialised literature for the region and other sub-Antarctic areas has been compiled by authors, including Ricker (1987), Adams (1994), Mendoza et al. (1996), Mendoza and Nizovoy (2000), Wiencke and Clayton (2002), Boraso et al. (2009), Hommersand et al. (2009), Ramírez (2010), Boraso de Zaixso and Zaixso (2013), Küpper et al. (2019) and Jofre et al. (2021). Taxonomic names were verified using the AlgaeBase database information webpage (Guiry and Guiry 2025). All specimens were pressed and preserved on herbarium sheets (Fig. 2d) and are currently housed at the Fundación Rewilding Herbarium in Puerto Varas, Chile. Macroalgal presence was then recorded across all photo-quadrats, with species-level identification performed wherever possible.

### Funding

This work was made possible by Fundación Rewilding Chile, a Chilean non-profit organisation, financially supported by an extensive philanthropic network. JK is supported by a Postdoc fellowship from CONICET.

## Geographic coverage

### Description

Inútil Bay is located in the Magallanes Region, at the southern tip of the Tierra del Fuego archipelago in South America (Fig. 1, Table 1).

### Coordinates

53°44.838' and 53°23.821' Latitude; 70°11.198' and 69°57.606' Longitude.

## Taxonomic coverage

### Description

**Figure 3.**
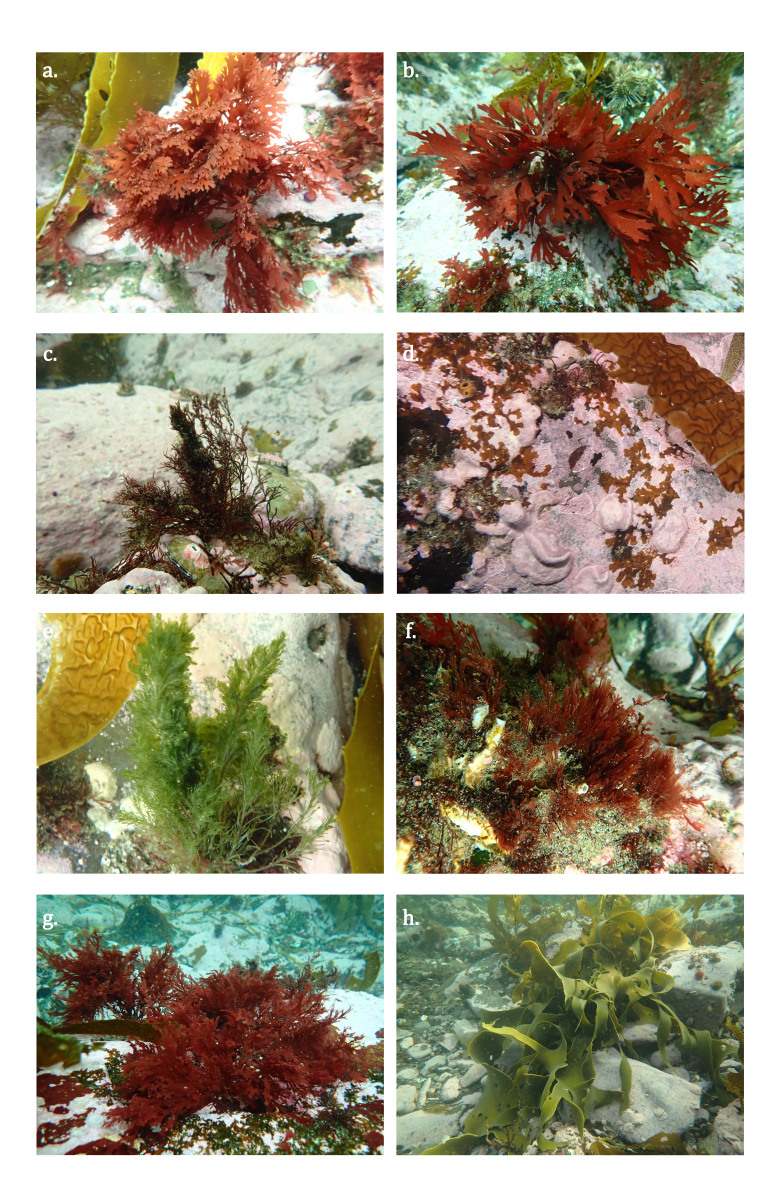
Examples of macroalgal species in Inútil Bay: **a** *Callophyllis variegata*; **b** *Callophyllis atrosanguinea*; **c** *Lophurella hookeriana*; **d** *Dictyota falklandica* and red encrusting coralline algae; **e** *Bryopsis australis*; **f** *Griffithsia antarctica*; **g** *Ptilonia magellanica*; **h** *Durvillaea antarctica*. Photographs: Julieta Kaminsky.

This study recorded a total of 72 macroalgal taxa: 32 in intertidal and 58 in subtidal habitats (Table 2). In the intertidal zone, we identified seven Chlorophyta, 10 Ochrophyta and 15 Rhodophyta. In the subtidal zone, nine Chlorophyta, 17 Ochrophyta and 32 Rhodophyta were found. The most frequently observed species, present at all sampling sites, included *Ptilonia magellanica* (Montagne) J. Agardh 1852, *Lophurella hookeriana* (J. Agardh) Falkenberg 1901, *Callophyllis variegata* (Bory) Kützing 1843 (Fig. 3) and the canopy-forming species *Macrocystis pyrifera* (Linnaeus) C. Agardh 1820, *Lessonia flavicans* Bory 1825 and *L. searlesiana* Asensi & de Reviers 2009. Additionally, some species are of particular interest. For example, two brown algal species were recorded: *Dictyota falklandica* Küpper, A.F.Peters, Asensi & De Clerck 2019 (Fig. 3d) and *Microzonia velutina* J. Agardh 1894 (Palacios et al. 2025). Other species, such as *Bryopsis australis* Sonder 1845, were rarely found (Fig. 3e).

## Temporal coverage

### Notes

Sampling took place 24-28 March 2025.

## Usage licence

### Usage licence

Creative Commons Public Domain Waiver (CC-Zero)

## Data resources

### Data package title

Sub-Antarctic subtidal and intertidal macroalgae in rocky ecosystems of Inútil Bay, Tierra del Fuego in Southern Patagonia

### Resource link

https://doi.org/10.15468/u5sxsr

### Alternative identifiers

https://www.gbif.org/dataset/6b96cd8f-e4a4-4837-84be-eac2f2701d72

### Number of data sets

1

### Data set 1.

#### Data set name

Sub-Antarctic subtidal and intertidal macroalgae in rocky ecosystems of Inútil Bay, Tierra del Fuego in Southern Patagonia

#### Data format

Darwin Core

#### Download URL

https://gbif-chile.mma.gob.cl/ipt/archive.do?r=macroalgas_bahia_inutil_2025&v=1.47

#### Description

This study provided the first description of the benthic marine macroalgal species richness found in the intertidal and subtidal environments of Inútil Bay, a sub-Antarctic ecosystem, located in the Magellanic Region of Chile (Kaminsky et al. 2025). Analysis of photo-quadrats led to the identification of 72 macroalgal taxa. This is the first study in the region to document macroalgal species richness supported by curated herbarium specimens and an image repository, expanding the phytogeographic characterisation of the region.

**Data set 1.** 

Column label	Column description
institutionCode	Institution holding the data, in this case "RewildingChile".
basisOfRecord	Type of record, in this case all records are "HumanObservation".
occurrenceID	An identifier for each occurrence, for example, “ChileInutilBay2025Site2subtidalPhotoquadratsTx001”, which indicates the first taxa recorded in the subtidal photo-quadrat sampling at Site 2.
eventID	An identifier for the set of information associated with an event (something that occurs at a place and time). May be a global unique identifier or an identifier specific to the dataset.
parentEventID	An identifier for the broader event that groups this and potentially other events.
locationID	The sampling site is indicated.
country	Name of the country, in this case "Chile".
stateProvince	Name of the county, district, or equivalent, in this case "MagellanicRegion".
year	Year of the event.
month	Month of the event.
day	Day of the event.
verbatimEventDate	The verbatim original representation of the date and time information for an event, in this case "fall 2025".
habitat	Description of the habitat (e.g. "subtidal", "intertidal").
samplingProtocol	The methods or protocols used, in this case macroalgae identified after diving collection or after processing photo-quadrats, indicated as "DivingCollection" or "Photoquadrats", respectively).
sampleSizeValue	A numeric value for a measurement of the size (time duration, length, area or volume) of a sample in a sampling event.
sampleSizeUnit	The unit of measurement of the size (time duration, length, area or volume) of a sample in a sampling.
verbatimDepth	Diving sampling depth.
decimalLatitude	Latitude in decimal degrees.
decimalLongitude	Longitude in decimal degrees.
geodeticDatum	Geodetic datum used, in this case "WGS84".
georeferencedBy	Person(s) who performed the georeferencing.
identifiedBy	Person(s) who identified the organism.
scientificName	Complete scientific name with authors.
kingdom	Kingdom name.
phylum	Phylum name.
class	Class name.
order	Order name.
family	Family name.
genus	Genus name.
specificEpithet	Specific epithet.
taxonRank	Taxonomic rank (e.g. species, genus).
taxonRemarks	Comments or notes about the taxon.
scientificNameAuthorship	Authorship of the scientific name.
establishmentMeans	Statement about whether an organism has been introduced to a given place and time through the direct or indirect activity of modern humans, in this case is indicated as "native" or "invasive".
type	The nature or genre of the resource, in this case "occurrence".
occurrenceStatus	A statement about the presence or absence of a taxon at a location.
rightsHolder	A person or organisation owning or managing rights over the resource, in this case "RewildingChile".
datasetName	The name identifying the dataset from which the record was derived.
eventDate	The date-time or interval during which an event occurred (e.g. "25-03-2025").
acceptedNameUsage	The full name, with authorship and date information if known, of the currently accepted taxon.
taxonomicStatus	The status of the use of the scientificName as a label for a taxon.
countryCode	The standard code for the country in which the sampling occurs, in this case "CL".
minimumDepthInMetres	The lesser depth of a range of depth below the local surface, in metres.
maximumDepthInMetres	The greater depth of a range of depth below the local surface, in metres.

## Figures and Tables

**Table 1.** Sampling sites with coordinates in Inútil Bay, Tierra del Fuego.

**Date**	**Site**	**Latitude / Longitude**
25-03-2025	1	53°44.838'S, 70°08.064'W
25-03-2025	2	53°44.633'S, 70°07.057'W
26-03-2025	3	53°23.821'S, 69°57.606'W
26-03-2025	4	53°27.370'S, 70°06.822'W
27-03-2025	5	53°42.861'S, 69°58.072'W
28-03-2025	6	53°28.012'S, 70°11.198'W

**Table 2.** Macroalgae species presence in Inútil Bay in intertidal and subtidal environments. *Species* or *Family* in bold indicate available specimens at the Fundación Rewilding Herbarium collection in Puerto Varas, Chile.

**Class**	**Order**	**Family**	**Species**	**Int**	**Sub**
**Phylum Chlorophyta**					
Ulvophyceae	Acrosiphoniales	Acrosiphoniaceae	*Acrosiphonia arcta* (Dillwyn) Gain 1912	x	x
	Bryopsidales	Bryopsidaceae	***Bryopsis australis* Sonder 1845**		x
		Codiaceae	***Codium dimorphum* Svedelius 1900**		x
			***Codium fragile* (Suringar) Hariot 1889**		x
			***Codium subantarcticum* P.C.Silva 1987**		x
		Derbesiaceae	*Derbesia marina* (Lyngbye) Solier 1846		x
	Ulvales	Ulvaceae	*Monostroma* Thuret 1854	x	
			*Ulva bulbosa* P.Beauvois 1805	x	
			*Ulva intestinalis* Linnaeus 1753	x	
			***Ulva lactuca* Linnaeus 1753**	x	x
			***Ulva taeniata* (Setchell) Setchell & N.L.Gardner 1920**	x	x
			***Ulva* Linnaeus 1753**	x	x
**Phylum Ochrophyta**					
Phaeophyceae	Dictyotales	Dictyotaceae	*Dictyota falklandica* Küpper, A.F.Peters, Asensi & De Clerck 2019		x
	Syringodermatales	Syringodermataceae	***Microzonia velutina* J.Agardh 1894**		x
	Desmarestiales	Desmarestiaceae	***Desmarestia confervoides* (Bory) M.E.Ramírez & A.F.Peters 1993**	x	x
			***Desmarestia ligulata* (Stackhouse) J.V.Lamouroux 1813**		x
	Ectocarpales	Ectocarpaceae	*Ectocarpus* Lyngbye 1819	x	x
		Acinetosporaceae	*Pylaiella littoralis* (Linnaeus) Kjellman 1872		x
		Adenocystaceae	***Adenocystis utricularis* (Bory) Skottsberg 1907**	x	x
			***Caepidium antarcticum* J.Agardh 1880**	x	x
		Chordariaceae	*Chordaria linearis* (Hooker f. & Harvey) Cotton 1915	x	
			***Corycus lanceolatus* (Kützing) Skottsberg 1921**		x
			***Leathesia difformis* (Fries) Areschoug 1847**	x	x
		Scytosiphonaceae	***Planosiphon nakamurae* M.Hoshino, M.E.Croce, Hanyuda y Kogame 2020**	x	x
			*Petalonia fascia* (O.F.Müller) Kuntze 1898	x	
	Fucales	Durvillaeaceae	***Durvillaea antarctica* (Chamisso) Hariot 1892**	x	x
	Laminariales	Laminariaceae	***Macrocystis pyrifera* (Linnaeus) C.Agardh 1820**		x
		Lessoniaceae	***Lessonia flavicans* Bory 1825**		x
			*Lessonia searlesiana* Asensi & de Reviers 2009		x
	Sphacelariales	Stypocaulaceae	***Halopteris obovata* (Hooker f. & Harvey) Sauvageau 1904**	x	x
		Cladostephaceae	***Cladostephus spongiosus* (Hudson) C.Agardh 1817**		x
**Phylum Rhodophyta**					
Bangiophyceae	Bangiales	Bangiaceae	*Pyropia/Porphyra* J.Agardh, 1899	x	
Florideophyceae	Balliales	Balliaceae	***Ballia callitricha* (C.Agardh) Kützing 1843**		x
	Bonnemaisoniales	Bonnemaisoniaceae	***Ptilonia magellanica* (Montagne) J.Agardh 1852**		x
	Ceramiales	Callithamniaceae	***Callithamnion* Lyngbye 1819**		x
			***Falklandiella harveyi* (Hooker f.) Kylin 1956**		x
		Ceramiaceae	***Ceramium diaphanum* (Lightfoot) Roth 1806**		x
			***Ceramium rubrum* C.Agardh, 1811 accepted as *Ceramium virgatum* Roth, 1797**	x	x
		Delesseriaceae	***Cladodonta lyallii* (Hooker f. & Harvey) Skottsberg 1923**		x
			***Delesseria* J.V.Lamouroux 1813**		x
			*Apoglossum* (J.Agardh) J.Agardh 1898		x
			***Heterosiphonia berkeleyi* Montagne 1842**		x
			***Hymenena laciniata* (Hooker f. & Harvey) Kylin 1924**		x
			***Pseudophycodrys phyllophora* (J.Agardh) Skottsberg 1923**		x
			***Phycodrys quercifolia* (Bory) Skottsberg 1922**		x
			***Schizoseris condensata* (Reinsch) R.W. Ricker 1987**		x
			***Schizoseris dichotoma* (Hooker f. & Harvey) Kylin 1924**		x
			***Schizoseris griffithsia* (Suhr) M.J.Wynne 1989**		x
		Rhodomelaceae	*Bostrychia intricata* (Bory) Montagne 1852	x	
			***Chondria secundata* (J.Agardh) De Toni 1903**		x
			***Lophurella hookeriana* (J.Agardh) Falkenberg 1901**		x
		Wrangeliaceae	***Griffithsia antarctica* Hooker f. & Harvey 1847**		x
		Corallinaceae	*Lithophyllum* Philippi 1837	x	x
			*Corallina officinalis* Linnaeus 1758	x	
			*Lithothamnion rugosum* Foslie 1900	x	x
			*Lithothamnion granuliferum* Foslie 1905	x	x
			*Lithothamnion* Heydrich 1897	x	x
			***Bossiella orbigniana* (Decaisne) P.C.Silva 1957**	x	
		**Ceramiales indet.**			x
	Nemaliales	Scinaiaceae	*Nothogenia fastigiata* (Bory) P.G. Parkinson 1983	x	
	Gigartinales	Areschougiaceae	***Acanthococcus antarcticus* Hooker & Harvey 1845**		x
		Gigartinaceae	***Iridaea cordata* (Turner) Bory 1826**		x
			*Iridaea tuberculosa* (J.D.Hooker & Harvey) Leister 1993	x	
			***Sarcopeltis skottsbergii* (Setchell & N.L.Gardner) Hommersand, Hughey, Leister & P.W.Gabrielson 2020**		x
			***Sarcothalia crispata* (Bory) Leister 1993**	x	x
		Kallymeniaceae	***Callophyllis variegata* (Bory) Kützing 1843**		x
			**Callophyllis variegata var. atrosanguinea (Hooker f. & Harvey) Dickie 1879**	x	
	Hildenbrandiales	Hildenbrandiaceae	*Hildenbrandia lecannellieri* Hariot 1887	x	
	Plocamiales	Plocamiaceae	***Plocamium cartilagineum* (Linnaeus) P.S.Dixon 1967**		x
			***Plocamium secundatum* (Kützing) Kützing 1866**		x
	Rhodymeniales	Rhodymeniaceae	***Rhodymenia coccocarpa* (Montagne) M.J.Wynne 2007**		x
Rhodophyta crustose indet.					x
